# Causal Inference in Multisensory Heading Estimation

**DOI:** 10.1371/journal.pone.0169676

**Published:** 2017-01-06

**Authors:** Ksander N. de Winkel, Mikhail Katliar, Heinrich H. Bülthoff

**Affiliations:** Department of Human Perception, Cognition, and Action, Max Planck Institute for Biological Cybernetics, Tübingen, Baden-Württemburg, Germany; University of Muenster, GERMANY

## Abstract

A large body of research shows that the Central Nervous System (CNS) integrates multisensory information. However, this strategy should only apply to multisensory signals that have a common cause; independent signals should be segregated. Causal Inference (CI) models account for this notion. Surprisingly, previous findings suggested that visual and inertial cues on heading of self-motion are integrated regardless of discrepancy. We hypothesized that CI does occur, but that characteristics of the motion profiles affect multisensory processing. Participants estimated heading of visual-inertial motion stimuli with several different motion profiles and a range of intersensory discrepancies. The results support the hypothesis that judgments of signal causality are included in the heading estimation process. Moreover, the data suggest a decreasing tolerance for discrepancies and an increasing reliance on visual cues for longer duration motions.

## Introduction

A large body of research shows that the Central Nervous System (CNS) processes multiple sensory signals on any particular environmental property in a fashion that is consistent with Bayesian inference, a strategy commonly referred to as Multisensory Integration (MI) [1–6]. According to this strategy, the CNS computes as the final estimate of a property the single value that is most likely given the sensory signals that represent it and any prior beliefs an individual may hold about the property. However, as our senses continuously process stimuli emanating from a multitude of events in the surrounding environment, the case where multiple signals actually represent a common property can be considered relatively rare; the majority of the signals should not be integrated. Consequently, it is plausible that the CNS somehow incorporates assessments of signal causality in the estimation of environmental properties [7, 8].

The idea that the CNS assesses signal causality is explicitly addressed in Causal Inference (CI) models of multisensory perception [9–11]. In CI models, a final estimate is constructed from two intermediate estimates. The intermediate estimates arise from different interpretations of the causality of the sensory signals. The first interpretation is that multiple signals share a common cause, where the MI strategy dominates the final estimate; the alternative is an interpretation of independent causes, where an individual channel determines the final estimate (Sensory Segregation; SS). A number of variants of the CI model have been proposed [11], that each construct a final estimate in a slightly different way: either by weighting and summing the intermediate estimates, or by applying a decision rule, where the weight or the probability of a specific choice is proportional to the probability of the respective causal structures.

In the majority of experimental studies on multisensory heading estimation, participants are presented with visual and inertial stimuli with minor discrepancies, assumed to be below the threshold of detection, or no discrepancies at all. Under these conditions, the general finding is that multisensory heading estimation is indeed consistent with the MI strategy (e.g., [12]). However, some findings violate the predictions of MI. For example, in [13], participants relied solely on one of the signals to estimate their heading. Other studies report multisensory integration for some, but not all, participants [14–16].

In previous work [17], we postulated that the dichotomy in experimental findings results from CI: whereas inertial information on self-motion is almost exclusively caused by self-motion (for exceptions, see e.g., galvanic stimulation, [18]), visually perceived motion can be caused by self-motion, object motion, or a combination thereof. Consequently, reports that are inconsistent with MI could imply that particularities of the stimuli led to an interpretation of independent causes. However, when we assessed the tenability of a CI model of multisensory heading estimation experimentally, the obtained data reflected MI regardless of the size of introduced discrepancies. This finding suggested that humans are essentially oblivious to discrepancies between visual and inertial heading. Although this conclusion is consistent with the finding that MI in heading estimation is robust for differences between the motion profiles of the visual and the inertial stimulus [19], it contrasts with earlier findings inconsistent with or partially supporting MI [13–16].

The most striking methodological difference between the reports consistent with MI and the aforementioned study by [13] concerns the characteristics of the motion profiles. Although the maximum acceleration was similar, at approximately 0.5m/s^2^, the motions presented in the latter study had translations and durations that were about an order of magnitude larger than the motions used in the former studies. These differences amounted to, respectively, a translation of seven meters over the course of almost ten seconds versus translations of a few decimeters in one to two seconds. This inspired the present hypothesis that the CNS does perform CI in the heading estimation process, but that the multisensory heading estimation process is affected by the characteristics of the motion profiles.

## Results

To test the hypothesis outline above, we performed two experiments (I and II). In both experiments, we used the Max Planck CyberMotion Simulator [20] to present participants with a large number (600-648) of unisensory visual-only and inertial-only, and multisensory visual-inertial motion stimuli, with headings ranging the full circle, and discrepancies up to ±90° (in the multisensory conditions). For each experimental trial, participants indicated the heading of self-motion using a pointer device. All motions followed a raised cosine bell velocity profile, and had durations of two, four, or six seconds. In experiment I (8 participants), maximum *velocity* was kept equal (0.30m/s) between motions of different duration; in experiment II (9 participants), maximum *acceleration* was kept equal (0.50m/s^2^). Differences between experiments were introduced to be able to dissociate potential effects of duration, velocity, and acceleration. Because motion profiles can be identified by their duration within experiments, we occasionally refer to a motion profile by its duration for convenience. For a complete overview of the task and stimuli, see section: Task and Stimuli. A video of simulator motion is available as supplementary material S1 Video.

From the responses obtained in the unisensory conditions, we determined whether patterns of bias and dispersion differed between motion profiles, and whether observations corresponded to the literature [17, 21, 22]; from the responses obtained in the multisensory conditions, we firstly determined whether either of a number of variants of a CI model could account for the data, and secondly whether there were differences in model parameters for different motion profiles.

To decide upon the best models of unisensory and multisensory heading estimation, we compared model Bayesian Information Criterion (BIC) scores [23]. This is a measure of model quality based on the model likelihood, number of observations, and the number of parameters. The model with the lowest BIC score is considered the best in an absolute sense. Differences in BIC scores of competing models, △BIC, between 0-2; 2-6; 6-10 are considered negligible, positive, and strong evidence, and >10 is considered decisive evidence.

### Unisensory Conditions

**Fig 1:**
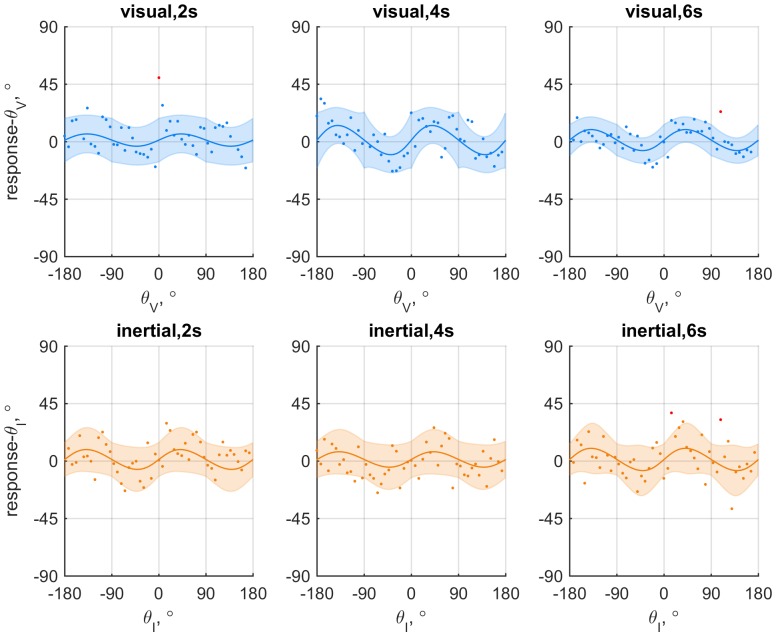
Data and fits for an exemplary participant (6) from experiment II split by experimental condition. The upper row shows the data for visual-only conditions; the bottom row shows the data for inertial-only conditions. Columns correspond to motion duration. Individual dots each represent the signed difference between the response and stimulus heading: red dots mark outliers (i.e., observation is more than three standard-deviations from *μ*(*θ*)). The colored lines show fit of the model where the parameters were allowed to vary between conditions. Shaded areas represent 95% confidence intervals.

Models that allow for periodic patterns in bias (constant error) and dispersion (variable error) in heading estimates were fitted to the responses obtained in the unisensory conditions. Both the bias and the dispersion were constructed as having a constant part and a part that varied periodically with heading, with a 90° period. We assessed if and how bias and dispersion of visual and inertial heading estimates were affected by characteristics of the motion profiles. Specifically, we compared the tenability of models in which the parameters that describe bias (*β*_0_, *β*_1_) and dispersion (*γ*_0_, *γ*_1_) were either kept constant between profiles or allowed to vary. The evidence in favor of the best fitting unisensory model for individual participants was always decisive, as compared to a null-model that did not include bias or variability of dispersions (△BIC = 72.1 − 770.0: see supplementary material S1 Table). As an illustration, Fig 1 shows for an individual participant the data and fits for a model where all parameters were allowed to vary.

**Fig 2:**
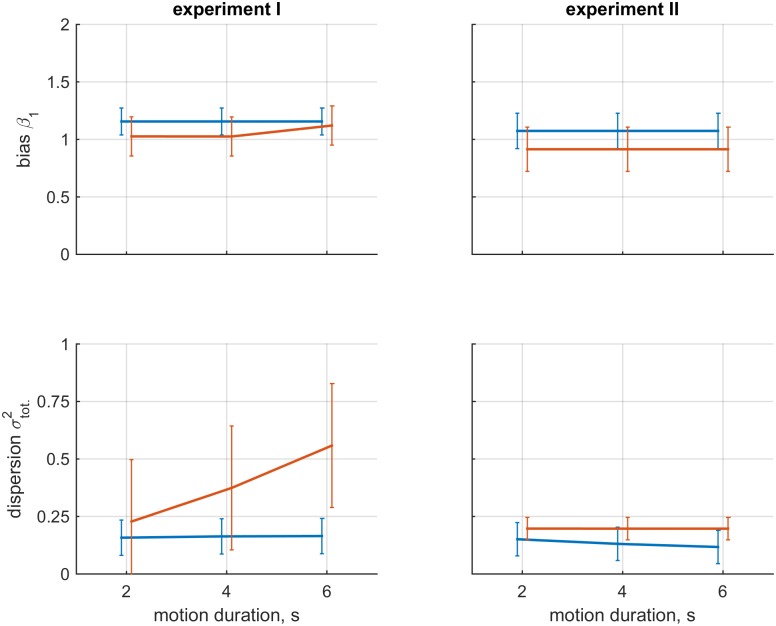
Visualization of findings on bias and dispersion aggregated over participants. The left column shows the data for experiment I; the right column shows the data for experiment II. The upper row shows the mean and standard deviation of parameter *β*_1_ for the different motion conditions; the bottom row shows the total dispersion. Blue and orange lines represent the visual-only and inertial-only conditions, respectively. The errorbars represent standard deviation.

In experiment I, a model in which the constant (*γ*_0_) and heading-dependent (*γ*_1_) parts of the dispersion of inertial heading estimates were allowed to vary was preferred for three out of eight participants, and a model in which only the constant part (*γ*_0_) varied was preferred for three others. The strength of the evidence favoring these models ranged between positive and decisive (△BIC = 4.06 − 18.02, median = 10.32). For the two remaining participants, there was no evidence for variability of bias or dispersion. For eight out of nine participants in experiment II, patterns of bias or dispersion were unaffected by motion duration. For the remaining participant, the constant part (*γ*_0_) of the visual dispersion varied for different motion durations (△BIC = 4.22). Findings on bias and total dispersion (see: S1 Appendix) of both experiments are visualized in Fig 2.

The finding of variability of dispersions for the majority of participants in experiment I suggested a systematic effect of motion characteristics on inertial heading estimation, and corresponded to participants’ subjective reports that task difficulty increased with motion duration. An assessment of total dispersion confirmed that the size of inertial dispersion increases for longer motion durations (△BIC = 2.708).

Apart from the observed effects of motion characteristics on dispersion in experiment I, the parameters that determine dispersion, *γ*_0_ and *γ*_1_, were generally positive and within the same order of magnitude, indicating that heading dependencies of variability in dispersion are similar in all conditions, with larger dispersions for headings further away from the cardinal axes.

The constant part of the bias (*β*_0_) amounted to 3.67° in experiment I, and 2.98° in experiment II. This parameter was included to account for factors such as the orientation of the participant relative to the simulator, and was kept equal in all conditions. For the visual conditions of experiment I and II, the median estimates of parameter *β*_1_ were 1.19 and 1.06; these averages were 1.02 and 0.88 in the inertial conditions. The corresponding peak sizes of the heading dependent part of the bias were 4.98° and 1.67° in the visual conditions, and 0.57° and 3.66° in the inertial conditions. There was considerable variability in the estimates of *β*_1_. In the visual conditions, the value of ranged between 0.85 − 1.33, corresponding to peak biases of 4.65° and 8.14°. In the inertial conditions, the estimates ranged between 0.67 an 1.76, corresponding to peak biases of 11.40° and 15.98°. Note that positive values of *β*_1_ indicate bias away from the cardinal axes, and negative values indicate bias towards the cardinal axes.

The estimated coefficients for all participants are provided in supplementary material S2 Table.

### Multisensory Conditions

The analysis of multisensory data consisted of a comparison of the tenability of a Multisensory Integration (MI) model, a Sensory Segregation (SS) model, and three variants of Causal Inference (CI) models: Model Averaging (MA), Model Selection (MS), and Probability Matching (PM) [11]. In the following we provide a concise description of the models. A more detailed description of the models can be found in section: Analyses of Multisensory Data and in [17].

The MI model is a circular analogue of the model proposed by [4]. According to this model, the CNS determines as a heading estimate the single heading angle that is most likely to have caused both the visual and inertial signals.

The SS model states that heading estimates are based upon either the visual or the inertial signal; the probability that heading estimates are based upon the visual signal is expressed by parameter *ξ*. *ξ* is treated as a free parameter, and takes on values between zero and one. Values of zero and one respectively correspond to complete dominance of the inertial and visual system.

In the CI models, heading estimates resulting from MI and SS are treated as intermediate estimates, and combined according to the probability that the visual and inertial signal share a common cause (P(C ∣ *x*_*V*_, *x*_*I*_)). This probability is proportional to the product of the likelihood of observing the specific pair of visual and inertial signals given that they have a common cause (P(*x*_*V*_, *x*_*I*_ ∣ C)), and a prior P(C) that reflects a tolerance for discrepancies independent of the individual sensory signals or any discrepancy between them. P(C) is treated as a free parameter that takes on a value between zero and one. When P(C) equals zero, there is no tolerance for discrepancies, and multisensory signals will always be segregated (i.e., the CI model behaves like the SS model). In contrast, when P(C) equals one, multisensory signals will always be attributed to a single cause (i.e., the CI model behaves like the MI model).

The variants of the CI model apply P(C ∣ *x*_*V*_, *x*_*I*_) in different ways. In the MA variant, P(C ∣ *x*_*V*_, *x*_*I*_) and its reciprocal serve as weights for the intermediate estimates according to MI and SS, respectively; in the MS variant, the final heading estimate is based upon the MI estimate when P(C ∣ *x*_*V*_, *x*_*I*_) is greater than 0.5, and based upon the SS estimate otherwise. Finally, in the PM variant, the final heading estimate is chosen at random from MI or SS, where P(C ∣ *x*_*V*_, *x*_*I*_) determines the probability of choosing MI.

On a more intuitive level, according to the MA model variant, the final heading estimate is a weighted average of the intermediate estimates according to the MI and SS strategies, with weights that correspond to the probability that the visual and inertial signal have a common, or independent causes. According to both the MS and PM variants, the final heading estimate is equal to *either* the MI or the SS estimate. The difference between these two variants is in how the choice is made. In the MS variant, the estimate with the most probable causal structure is chosen as the final estimate, whereas in the PM model, the final estimate is chosen at random between the two alternatives, with a probability of deciding on either the MI or the SS estimate that is equal to the probability of the corresponding causal structures.

**Fig 3:**
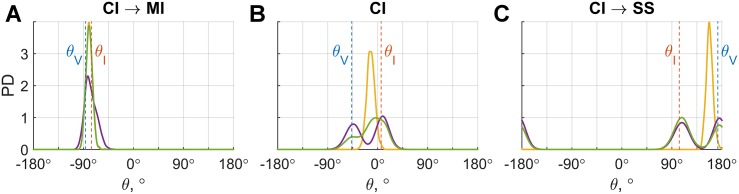
Visualization of probability densities of the response for hypothetical experimental trials, according to the MI(yellow), SS(purple), and CI-MA(green) models. The dashed vertical line in blue marked *θ*_*V*_ represents visual heading angle; the orange line marked *θ*_*I*_ represents inertial heading angle. The left panel illustrates that the CI-MA model behaves very similar to the MI model for small discrepancies; the middle panel shows that the CI-MA model averages between the MI and SS model for an ‘intermediate’ discrepancy; and the right panel shows that the CI-MA model behaves very similar to the SS model for large discrepancies. The likelihood of a response according to either model corresponds to the height of the associated density function at the response angle.

Because the models and their variants differ in the way responses are constructed, there are also differences between the density functions that represent the likelihood of specific responses according to each of these models. We can evaluate which model corresponds best to the data by comparing these likelihoods. As an illustration of the behavior of the main models, Fig 3 shows the densities according to the MI, SS, and MA model for hypothetical experimental trials with a small, intermediate, and large visual-inertial discrepancy.

We assessed whether the characteristics of multisensory processing depended on characteristics of the motion profiles by determining if the overall model quality improved between fitting either a single pair of *ξ* and P(C) parameters for all motion profiles, or fitting three pairs of *ξ* and P(C) parameters; one pair for each profile. In the latter case, the models’ and parameters’ association to specific conditions is indicated by the subscript ‘*T*’, which represents the duration of the motion profiles.

Note that allowing the prior P_*T*_(C) to vary between motion duration conditions implies that it cannot be interpreted as an a-priori belief about signal causality at stimulus onset: participants were unaware of the motion characteristics at stimulus onset because stimuli were presented in random order, and beliefs could therefore not be adapted to the conditions at that exact moment. Instead, we interpret P_*T*_(C) as reflections of the tolerance for discrepancies after *T* = 2, 4 and 6s of motion, which is independent of the size of the discrepancy between sensory signals.

Due to their three dimensional nature, the raw data do not lend themselves well for visualization. Plots of the response bias (relative to visual heading angle) as a function of the size of the discrepancy between the visual and inertial heading angle are available as supplementary material S1 Fig.

#### Model Comparisons

Model comparisons for experiment I favored the CI-MA model for six out of eight participants. Compared to the best fitting alternative model (i.e., MI or SS), the evidence favoring a CI model was decisive in five out of these six cases (△BIC = 12.8 − 125.4), and strong in the remaining case (pp. 8; △BIC = 9.9). Data for the two remaining participants were best explained by the *SS* model.

Model comparisons in experiment II favored a CI model for eight out of nine participants. Five of these eight cases were in favor of the MA model; three in favor of the PM model. The evidence favoring CI models was decisive in six out of eight cases (△BIC = 10.7 − 116.8), strong in one case (△BIC = 8.2), and positive in the remaining case (△BIC = 2.3). Data for the remaining participant were best explained by the *SS* model.

Combined over all participants, the MA model provided the best explanation of the data. The evidence was decisive, compared to the MI and SS model (△BIC = 21869.8, and △BIC = 440.6, respectively). All BIC scores can be found in supplementary material S3 Table; all obtained parameter estimates are presented in supplementary material S4 Table.

#### Multisensory effects of motion characteristics

For three of the participants from experiment I for whom the CI model provided the best fit, overall model fit was further improved by fitting the model separately for the different motion duration conditions. For these participants, the median estimates for P_*T*_(C) were 0.96, 0.61, and 0.11, for *T* = 2, 4, and 6s, respectively; the median values of parameter *ξ*_*T*_ were 0.32, 0.60, and 0.68.

In experiment II, the evidence favored parameter variability in two cases. For these cases, the median estimates of P_*T*_(C) were 0.95, 0.93, and 0.39, for *T* = 2, 4, and 6s, respectively; the median values of parameter *ξ*_*T*_ were 0.67, 0.99, and 1.00.

**Fig 4:**
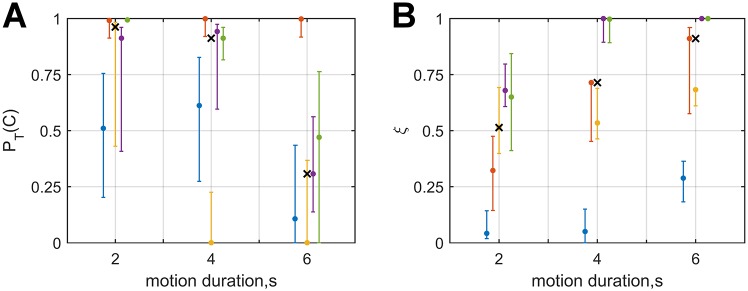
Estimates of the tolerance for discrepancies prior P_*T*_(C) (left panel) and reliance on the visual estimate *ξ*_*T*_ (right panel) for participants of both experiments for whom there was evidence that the parameters varied with motion duration, plotted against motion duration. Data points in blue, orange, yellow, purple, and green correspond to experiment-participant I3, I7, I8, II4, and II5, respectively. Errorbars represent the parameters’ 95% confidence intervals, and the x-markers represent the median estimate for each P_*T*_(C) and *ξ*_*T*_.

Combined over experiments, the value of parameter P_*T*_(C) for the *T* = 6s condition was generally lower than in the other two motion duration conditions, suggesting a decreasing tolerance for discrepancies independent of unisensory effects (Fig 4, left panel). Estimates of parameter *ξ*_*T*_ consistently increased between the *T* = 2s and *T* = 6s conditions, suggesting increasing reliance on the visual heading angle (Fig 4, right panel). These effects also hold when parameter estimates for the other participants are included. See supplementary material S4 Table for an overview of all the parameter estimates for multisensory models.

**Fig 5:**
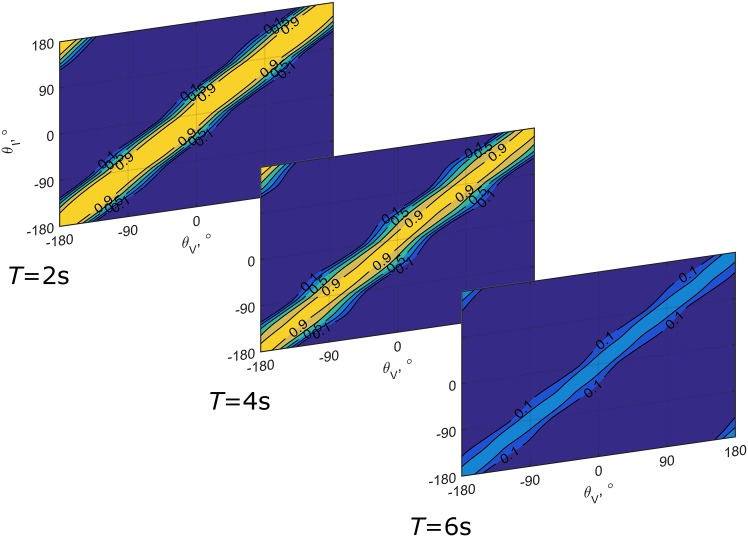
Visualization of the posterior probability that visual and inertial heading stimuli have a common cause P_*T*_(C ∣ *x*_*V*_, *x*_*I*_), for each condition separately. The figure was created using the median values of P_*T*_(C) from Fig 4. Yellow represents a high probability; blue a low probability. The variability of the band’s width results from bias in unisensory heading estimates and heading dependency of the dispersions.

Fig 5 provides a visualization of how the probability that any pair of visual and inertial heading signals will be attributed to a common cause, P(C ∣ *x*_*V*_, *x*_*I*_), varies as a function of the different values of P_*T*_(C) obtained in the experiment, taking into account unisensory effects. The decreasing width of the band for which the probability of a common cause is relatively high is indicative of a declining tolerance for discrepancies as stimulus duration increases; the variability of the width of the band for different heading angles results from heading dependencies of unisensory biases and dispersions.

## Discussion

In previous work [17], we evaluated the hypothesis that the Central Nervous System (CNS) performs Causal Inference (CI) in multisensory heading estimation. The obtained results, however, did not support the hypothesis, as the data could be explained equally well by the simpler Multisensory Integration (MI) model. Here we hypothesized that the lack of support for the CI model was due to the characteristics of the motion profiles. We assessed how multisensory heading stimuli were processed for motions with different durations, maximum velocities, and accelerations. In the following sections, we discuss the observations made on unisensory and multisensory heading estimation.

### Unisensory Heading Estimation

In Experiment I, we observed that the dispersion of inertial heading estimates increased for longer duration motions, whereas this effect was absent in Experiment II. This result can be explained by considering that the otoliths of the vestibular system, which are the main contributor to perception of translational motion [24, 25], respond to acceleration: in Experiment I, maximum velocity was kept constant between motions of different durations. Consequently, the motions’ maximum acceleration was lower for longer duration motions, and therefore the stimulus intensity was lower as well. In terms of Signal Detection Theory [26], low intensity signals are more difficult to distinguish from background noise than high intensity signals. This implies that misinterpretations of heading were more likely for the longer duration motions, resulting in larger dispersions. In contrast, in Experiment II, where maximum acceleration was kept constant, dispersion of heading estimates was found to be constant for all motion durations.

Apart from the variability of dispersion observed in experiment I, the findings on dispersion are consistent with the literature: inertial dispersion is larger than visual dispersion, and patterns in dispersion show that the smallest values occur around the cardinal axes, with values increasing as the heading angle deviates away from these axes [17, 21, 22, 27].

In a number of recent studies, both visual and inertial heading estimates were found to be biased away from the cardinal axes [21, 22, 27]. The present data show that visual heading estimates are on average also biased away from the fore-aft axis, in both experiments; inertial heading estimates were biased away from the cardinal axes in experiment I and biased towards the cardinal axes in experiment II. However, individual results show that there is considerable variability in the direction and size of the bias between participants. Such variability was also observed in our previous study, using the same visual environment [17]. Biases in heading estimation are thought to result from non-uniformity of preferred directions of neurons at the otolith epithelium [28] and in area MSTd of the visual cortex [29]. The observed variability in biases could therefore suggest that the distribution of preferred directions in these neural populations varies between individuals.

Interestingly, the absence of a decline in the size of sensory bias for longer duration motions supports the notion that biases in heading estimation result from non-uniformity in the distribution of preferred directions rather than a-priori preferences for particular heading directions [22]; if bias in heading estimation was due to a-priori beliefs favoring particular directions, the relative impact of these beliefs on the resultant estimate should decline for longer duration motions, as accumulating sensory evidence devoid of bias would eventually outweigh any prior beliefs.

### Multisensory Heading Estimation

The present analysis of multisensory data can be divided into two parts: in the first part, we assess whether the responses obtained in the multisensory conditions conform best to MI, SS, or CI; in the second part of the analysis, we assess whether characteristics of the motion profiles affect multisensory processing.

The first part of the analysis is nearly identical to [17]. However, whereas the conclusion in that study was that multisensory responses were best explained by the MI model (given an overall △BIC = 47.76, compared to CI), the present results provide decisive evidence in favor of the CI-MA model (△BIC = 21869.8 vs. MI, and △BIC = 440.6 vs. SS). The CI model is an alternative to multisensory interpretations (e.g., [9, 30–34]) of the robust cue integration model that was originally proposed to model intrasensory cue combination in visual perception of depth (e.g., [35–38]); hereafter referred to as interaction-prior (IP) model [9, 33]. IP models also allow responses to range between sensory segregation and forced fusion, but do so by making use of multidimensional priors that represent the strength of coupling between sensory systems as a function of discrepancy between the cues (e.g., [31, 39, 40]. Although it is possible to cast the CI model as an IP model [32], there are conceptual differences: first, IP models do not assume that there are alternative causal structures, and do not make predictions on judgments of causality; and second, the IP used in these models must be estimated from the data or designed by the experimenters. In the present case, the IP should essentially take the shape of the probability maps presented in Fig 5 [32, 33]. In a comparison of the performance of a CI model and IP models proposed by Roach et al. [39] and Bresciani et al. [31] for audio-visual perceptions, Beierholm et al. [32] found that a CI model performs slightly better than the IP model alternatives. Moreover, Rohe and Noppeney [41] recently identified a cortical hierarchy that appears to perform CI: segregated internal representations of multisensory stimuli exist in unisensory cortical areas; forced fusion occurs in the posterior intraparietal sulcus; and a combination of the intermediate estimates according to the probability of the respective causal structures is constructed in the anterior intraparietal sulcus. Recent literature thus tends to support the notion of CI, and we interpret the present evidence as support for the notion that the CNS incorporates assessments of causality in multisensory heading estimation.

In the second part of the analysis, we split the data up by motion profile and fitted the models separately. This was done to determine whether multisensory processing is affected by characteristics of the motion profiles, apropos the hypothesis. A comparison of the quality of the fits provided evidence that splitting up the data improved the fit for 5 out of 14 participants. There was no distinct difference in the number of participants for whom the evidence favored separate models between experiments I and II (3/6, resp. 2/8).

The fact that CI was favored regardless of evident differences between motion profiles suggests that the experimental manipulations were more powerful than in the previous study. This finding might reflect the fact that there was a higher relative incidence of stimuli with large discrepancies in the present study than in [17], because intersensory discrepancies were drawn from a uniform distribution rather than from a normal distribution, with the same effective extremes of ±90°.

For the participants for whom the evidence favored separate fits, the strength of the evidence was strong in two cases, and decisive in three. Consequently, even though explicit support for differences in multisensory processing was only found for a minority of participants, the strength of the evidence warrants further consideration of the results. The obtained estimates of the P_*T*_(C) parameters are shown in Fig 4. We interpret these parameters as reflections of the tolerance for discrepancies acting on the heading estimation process at time *T*. The effect of the value of this parameter on the posterior belief that a visual and an inertial heading have a common cause P_*T*_(C ∣ *x*_*V*_, *x*_*I*_) is visualized in Fig 5. As the figure shows, the width and height of the band of combinations for which the strength of this belief is relatively high decreases for increasing motion durations due to the value of P_*T*_(C). The value of this parameter decreases with motion duration. A decreasing tolerance for discrepancies could reflect accumulation of evidence on signal causality over time. Such a mechanism in latent decision making would be consistent with findings on evidence accumulation in human and animal overt decision making (e.g., [42–45]). Unfortunately, the current data are not suitable to determine the characteristics of such an hypothetical updating mechanism, as we do not know the rate at which this evidence accumulates, nor do we have access to intermediate internal representations.

For six second motions, the belief that visual and inertial heading stimuli have a common cause can be considered weak even for stimulus combinations that yield very similar internal heading representations. The effect is apparent in Fig 5: for the six second duration conditions, the probability of an interpretation of common causes is low even when visual and inertial heading angles are identical. This suggests that visual stimuli may not have been considered as reflecting self-motion, which also resembles the findings of [13], who reported that participants segregated congruent visual and inertial heading cues completely for even longer duration motions (9.3s).

An alternative explanation of the findings on P_*T*_(C) and *ξ*_*T*_ that is consistent with this finding might be found in the dynamics that have been reported for the visual and inertial perception of self-motion. It has been suggested that visually induced perceptions of self-motion (i.e., ‘vection’, see e.g.: [46–48]) are consistent with predictions based on *low-pass* frequency filtering of the visual input([49]). In other words, the CNS segregates visually perceived motion based on frequency content: the low frequency component is attributed to self-motion, and the high frequency component to object motion. In contrast, perceptions of translation resulting from inertial stimulation are consistent with segregation based on *high-pass* frequency filtering of otolith afferents (e.g., [49–54]): the high frequency component of the gravito-inertial acceleration is attributed to translation, whereas the low-frequency component is attributed to body tilt relative to gravity. The cut-off frequencies of the inertial high-pass filter [51, 53–57], and the visual low-pass filter [49] are estimated to be approximately 0.5Hz –although the exact value of the estimates varies between studies and individuals, from approximately 0.1 to 1Hz. The cut-off frequency indicates a boundary above or below which the output of a filter begins to be attenuated (for a low-pass and high-pass filter, respectively). Consequently, the range of motion frequencies for which both the visual and inertial systems contribute perceptions of self-motion may only partially overlap; near the cut-off frequency. This limited overlap could explain the observed decrease in the tendency to integrate multisensory information with motion duration, as well as the dichotomy in the literature: if we interpret the duration of the motions as the reciprocal of frequency, observations appear to support MI when motion frequencies are such that both systems are thought to yield perceptions of self-motion (i.e., close to the cut-off), whereas observations appear to oppose MI for lower frequencies, such as in [13] and the 6s condition of the present experiment. It could also explain the data of the three participants for whom the SS model was favored: the dynamics of the individual sensory systems may have been such that there was no overlap for the range of durations/frequencies tested.

Finally, it is interesting to note that in the study by De Winkel et al. [13], where participants appeared to segregate congruent visual-inertial cues, the visual stimulus was presented monoscopically, and consisted of motion through a black virtual environment populated with white circles; in the present study, the visual stimuli were presented stereoscopically, and consisted of limited lifetime random dots in an environment with a blue sky, and a grass like ground plane, with the horizon approximately at eye height. The present stimuli thus provided scaling information that allowed participants to relate the velocity specified in the visual flow magnitude to the inertial velocity, which was not possible in the earlier study. Consequently, the observed segregation of congruent stimuli in that study may also have been caused by perceptions of intersensory discrepancy in motion amplitude, which would imply that CI is also performed on the basis of discrepancies in other stimulus dimensions. This notion is consistent with findings from literature on vehicle simulation, which report on specific deterministic tolerances for discrepancies between the amplitude of visual and inertial motions (e.g., [58–60]).

We conclude that the current results provide supporting evidence for the hypothesis that the CNS includes judgments of signal causality in heading estimation. Moreover, specific findings in a subset of participants suggest that multisensory heading estimation for the purpose of self-motion perception might result from a complicated interplay between the dynamics of unisensory processing and multisensory strategies.

## Materials and Methods

The experimental paradigm, equipment and analyses are adapted from those used in [17]. Descriptions of the changes implemented in the present analysis to account for effects of motion characteristics are provided below. For a more detailed account on the derivation of the models, please refer to the aforementioned study.

### Ethics statement

The experiment was conducted in accordance with the Declaration of Helsinki. All participants gave their written informed consent prior to participation. The experimental protocol and consent forms were approved by the ethical commission of the medical faculty of the Eberhard-Karls University in Tübingen, Germany.

### Participants

Eight participants (5F; aged 19-34, mean 27.3) were recruited to take part in experiment I; nine participants (6F; aged 19-34, mean 25.8) were recruited to take part in experiment II. Two participants participated in both experiments: participants 1 and 7 in Experiment I are the same participants as 3 and 2 in Experiment II. Except for these two, participants were naïve to the purposes of the study. The participants all reported minimal susceptibility to motion sickness and claustrophobia, and no history of any intestinal, neurological, or vestibular illnesses. Due to safety regulations of the motion simulator, participation was only allowed for people between 18-65 years old, weighing up to 90kg, and measuring at most 1.95m.

### Task and Stimuli

In both experiments, stimuli consisted of presentations of horizontal linear translational motions with various headings, in a visual-only, an inertial-only and a combined visual-inertial condition. The stimuli were presented using the Max Planck Institute CyberMotion Simulator facility [20]. Visual stimuli showed movements through a three dimensional environment with a ground plane and limited-lifetime particles, presented stereoscopically using a dual projection system in conjunction with stereo glasses (Infitec^®^GmbH, Ulm, Germany, model INFITEC^®^Premium Glasses). Inertial stimulation was achieved by translating participants seated in the simulator cabin along the simulator’s linear track. To achieve different headings, the simulator cabin was oriented at different angles relative to the linear track. A video of simulator motion is available as supplementary material S1 Video.

For all motions, the motion profile followed a raised cosine bell in velocity, specified as

$$\begin{array}{c} v\left(t\right)=\frac{v_{max}}{2}\left(1-\cos\frac{2\pi t}{T}\right) \end{array}$$ (1)

where *t* is time and *v*_*max*_ is maximum velocity. *T* represents the period of the motion profile, which took values of 2, 4 and 6s. The translations, velocities, and accelerations of the motions used in experiment I and II are presented in Table 1.

**Table 1:** Motion duration *T*, translation *x*, maximum velocity *v*_*max*_, and maximum acceleration *a*_*max*_, for experiment I and II.

	experiment I			experiment II		
*T*	*x*, m	*v*_*max*_, m/s	*a*_*max*_, m/s^2^	x, m	*v*_*max*_, m/s	*a*_*max*_, m/s^2^
2	0.30	0.30	0.47	0.32	0.32	0.50
4	0.60	0.30	0.24	1.27	0.64	0.50
6	0.90	0.30	0.16	2.87	0.96	0.50

In the unisensory visual-only and inertial-only conditions of experiment I(cf. experiment II), 72(50) stimuli were presented for each of the three motion durations, with headings evenly spaced between ±180°, in 5°(7.2°) steps.

In the combined visual-inertial conditions, the stimuli were 72(100) visual motions with headings evenly spaced between ±180°, in 5°(3.6°) steps, paired with inertial motions with headings that had a discrepancy from the visual heading randomly drawn from a uniform distribution ranging between ±90°.

Participants were instructed to estimate the heading of self-motion on every experimental trial using a pointer device. This device consisted of a 20cm stainless steel rod mounted to a potentiometer. One end of the rod was covered by 5cm of black heat shrink tubing; the other end was to be interpreted as an arrow’s head and was to be pointed in the direction of the motion. The pointer device was free of discontinuities, and provided a <0.1° resolution. Because visual-only stimuli do not necessarily induce a sensation of self-motion, participants received the additional instruction to indicate the heading suggested by the visual stimulus for visual-only trials when no self-motion was perceived. For each participant, a total of 648(600) stimuli were presented in randomized order. Stimulus presentations were divided in blocks of 30 minutes, with compulsory breaks of at least 15 minutes in between. The experiment consisted of 3(2) 3(4)-hour sessions, which were completed over the course of 3(2) days.

### Data Analyses

The model fitting procedures were performed for each participant individually. To assess general effects of motion profile characteristics on unisensory and multisensory heading estimation, we tested for trends in the observations made for individual participants.

#### Analyses of Unisensory Data

Unisensory visual and inertial heading estimation were described using a model that can account for periodic patterns of bias and dispersion as these are reported in the literature [17, 21, 22, 29].

The output of each sensory system is modeled as a realization *x* of a von Mises distributed random variable with mean *μ* and concentration parameter *κ*:

$$\begin{array}{c} R=x∼M(\mu ,\kappa ) \end{array}$$ (2)

with

$$\begin{array}{c} \mu =\beta_{0}+\text{atan}\left(\beta_{1mT}\sin\theta ,\cos\theta \right) \end{array}$$ (2a)

$$\begin{array}{c} \kappa =\gamma_{0mT}-\gamma_{1mT}\left|\sin2\theta \right| \end{array}$$ (2b)

here *θ* is the stimulus’ heading, *β*_0_, *β*_1*mT*_, *γ*_0*mT*_, and *γ*_1*mT*_ are free parameters, and atan represents the four-quadrant inverse tangent function. The subscript *m* refers to the sensory modality, which was visual or inertial, and the subscript *T* refers motion duration, which was 2, 4 or 6s. The final responses to the unisensory stimuli, *R*, are assumed to be equal to *x*.

Parameter *β*_0_ was included to account for any small constant error that could arise due to factors such as a small offset in the position of a participant with respect to the simulator. This parameter was assumed to be constant for all conditions.

By either keeping the other six parameters (*β*_1_, *γ*_0_, *γ*_1_ times two sensory modalities) constant or allowing them to vary for the different profiles, we end up with a total of 2^6^ = 64 different models. Each model was fitted to the data using the method of Maximum Likelihood. We exhaustively compared model fits by means of the Bayesian Information Criterion (BIC) scores [23]. For each participant, we chose as the final unisensory model the model with the lowest BIC score, for which the evidence was at least positive compared to alternative models with fewer parameters. Data points more than three standard deviations away from the mean (i.e., *μ*(*θ*)) were considered outliers in the unisensory conditions and excluded from the analysis.

To assess whether the characteristics of the motion profile affected the bias or dispersion of unisensory heading estimates, we looked for general trends in the parameters obtained for all the participants. Trends in the bias were assessed by testing whether parameter *β*_1_ differed between profiles. Because dispersion is characterized by the combination of *γ*_0_ and *γ*_1_, and varies with heading angle, we needed a condensed measure that takes both parameters into account. Therefore, we calculated a total variance $\sigma_{tot.}^{2}$ for the different profiles (see supplementary material S1 Appendix). The values of $\sigma_{tot.}^{2}$ obtained were subsequently tested for differences between profiles.

#### Analyses of Multisensory Data

Heading estimation for multisensory stimuli was modeled according to a Multisensory Integration (MI) model, a model that reflects Sensory Segregation (SS), and three variants of a Causal Inference (CI) model [17]. The MI and SS models form extremes of a spectrum, for which multisensory signals are either completely integrated or segregated; the CI models come in between these extremes. Responses to multisensory stimuli were compared to model predictions to determine which model could best account for the observations. The CI models tested here are based upon the model originally proposed by [9], and the adaptations of that model that account for different decision strategies by [11]; the present versions are further modified to account for the circular nature of heading (see: [17]).

In each multisensory model, final heading estimates are constructed from the internal representations *x*_*V*_, *x*_*I*_ of the visual and inertial stimulus on the one hand, and knowledge of the size of their respective noises *κ*_*V*_, *κ*_*I*_ (i.e., dispersions) on the other. Because internal representations cannot be directly measured, we used the fitted unisensory models (see section: Analyses of Unisensory Data) to simulate them. Consistent with the unisensory models, *x*_*V*_ and *x*_*I*_ are realizations of von Mises distributed random variables with a heading dependent bias and noise. For each experimental trial, 1000 *x*_*V*_, *x*_*I*_ pairs were generated, from which final estimates were constructed according to each multisensory model. The likelihood of actual responses was subsequently derived from kernel density estimation [61] on the simulated data. Data points that differed more than ±90° from either the visual or inertial stimulus were considered outliers in the multisensory conditions.

We assume that the CNS is unaware of the relationship between the size of the noise and the stimulus’ heading angle. This assumption may be justified by the fact that if this relationship *was* known to the CNS, this would provide information which could be used to avoid bias. However, as bias in heading estimation is a common experimental finding [17, 21, 22, 62–65], it seems reasonable to assume that this relationship is unknown to the CNS. Consequently, the relationship between the *κ*_*V*_, *κ*_*I*_ and physical stimuli is not included in the multisensory models. For each experimental trial, the values of *κ*_*V*_, *κ*_*I*_ given by the unisensory models were passed to the multisensory models. Because the values of *κ*_*V*_, *κ*_*I*_ were equal for the 1000 simulations per experimental trial, they only enter the equations for the CI models (below) implicitly.

The response according to the MI model *R*_*MI*_ is the Maximum-A-Posteriori (MAP) heading estimate given the sensory signals and prior beliefs about the probability of different heading angles. Assuming a uniform prior on heading angle, *R*_*MI*_ can be expressed as

$$\begin{array}{c} R_{MI}=\mathrm{Arg}\left(\kappa_{V}e^{ix_{V}}+\kappa_{I}e^{ix_{I}}\right), \end{array}$$ (3)

where the function Arg refers to the argument of a complex number, yielding an angle.

According to the SS model, the response *R*_*SS*_ is either the estimate carried by the visual or the inertial channel, chosen at random:

$$\begin{array}{c} R_{SS}=\{\begin{array}{cc} x_{V}, & \text{with}\;\text{probability}\;\xi \\ x_{I}, & \text{with}\;\text{probability}\;1-\xi \end{array} \end{array}$$ (4)

where *ξ* represents the probability that the visual channel dominates. *ξ* was treated as a free parameter.

According to the CI models, the CNS constructs a final estimate from *R*_*MI*_ and *R*_*SS*_, while taking into account the posterior probability P(C ∣ *x*_*V*_, *x*_*I*_) that the unisensory signals have a common cause given the actual sensory signals. P(C ∣ *x*_*V*_, *x*_*I*_) is calculated as

$$\begin{array}{c} P\left(C|x_{V},x_{I}\right)=\frac{P\left(x_{V},x_{I}|C\right)P\left(C\right)}{P(x_{V},x_{I})} \end{array}$$ (5)

The likelihood P(*x*_*V*_, *x*_*I*_ ∣ C) represents the probability of observing the pair of visual and inertial signals *x*_*V*_, *x*_*I*_ given that the signals have a common cause. The prior P(C) is interpreted as a tolerance for discrepancies, isolated from the sensory information. The probability of the observations P(*x*_*V*_, *x*_*I*_) is a normalizing factor that ensures that the posterior is a proper probability density. It is calculated as

$$\begin{array}{c} P\left(x_{V},x_{I}\right)=P\left(C\right)P\left(x_{V},x_{I}|C\right)+\left(1-P\left(C\right)\right)P\left(x_{V},x_{I}|\bar{C}\right) \end{array}$$ (6)

In each version of the CI model, knowledge of probability P(C ∣ *x*_*V*_, *x*_*I*_) is applied in a different way. In the Model Averaging (MA) variant, intermediate estimates *R*_*MI*_ and *R*_*SS*_ are weighted according to P(C ∣ *x*_*V*_, *x*_*I*_) and the complement of this probability $P(\bar{C}|x_{V},x_{I})$, respectively:

$$\begin{array}{c} R_{\text{MA}}=\mathrm{Arg}\left(P\left(C|x_{V},x_{I}\right)e^{iR_{MI}}+P\left(\bar{C}|x_{V},x_{I}\right)e^{iR_{SS}}\right) \end{array}$$ (7)

In the Model Selection variant (MS), the final estimate equals *R*_*MI*_ or *R*_*SS*_, depending on which causal structure is more likely: P(C ∣ *x*_*V*_, *x*_*I*_) or $P(\bar{C}|x_{V},x_{I})$, respectively:

$$\begin{array}{c} R_{\text{MS}}=\{\begin{array}{cc} R_{MI}, & \text{if}\;P\left(C|x_{V},x_{I}\right)\geq \frac{1}{2}\\ R_{SS}, & \text{if}\;P\left(C|x_{V},x_{I}\right)<\frac{1}{2} \end{array} \end{array}$$ (8)

According to the Probability Matching (PM) variant, the final estimate also equals *R*_*MI*_ or *R*_*SS*_, but is chosen at random with probabilities matching P(C ∣ *x*_*V*_, *x*_*I*_) and $P(\bar{C}|x_{V},x_{I})$.

$$\begin{array}{c} R_{\text{PM}}=\{\begin{array}{cc} R_{MI}, & \text{with}\;\text{probability}\;P(C|x_{V},x_{I})\\ R_{SS}, & \text{with}\;\text{probability}\;P(\bar{C}|x_{V},x_{I}) \end{array} \end{array}$$ (9)

In the MA, MS, and PM models, parameters P(C) and *ξ* are equal for the different motion profile conditions. Consequently, these models cannot account for differences in multisensory responses between conditions that are not explained by characteristics of unisensory processing. To assess whether there are differences in multisensory processing, we assessed if the overall fit of the MA, MS, and PM models improved when the models were fitted to the different conditions individually; thus fitting three pairs of P(C) and *ξ* parameters rather than one. We indicate the condition associated with a parameter estimate by adding a subscript *T*, P_*T*_(C) and *ξ*_*T*_, where the subscript relates to the motion profile’s duration *T*, with *T* = 2, 4 and 6s. We interpret P_*T*_(C) as reflections of the tolerance for discrepancies after *T* = 2, 4 and 6s of motion, independent of information on discrepancy carried by the sensory signals themselves.

## Supporting Information

S1 FigResponse bias as a function of discrepancy.Plots of the bias in responses relative to visual heading angle (dots) as a function of discrepancy between visual and inertial heading angle. Dots with different colors represent data from different participants. The horizontal blue line indicates a perfect theoretical correspondence between responses and the visual heading angle; the diagonal orange line indicates a perfect theoretical correspondence between responses and the inertial heading angle. Discrepancy is defined as the angular difference between visual and inertial heading angle; bias is defined as the angular difference between the visual heading angle and the response. Respectively, the corresponding calculations are: $\mathrm{Arg}(e^{i\theta_{V}}/e^{i\theta_{I}})$, and $\mathrm{Arg}(e^{i\theta_{V}}/e^{i\theta_{R}})$.(PDF)

S1 Table Unisensory BIC scores. BIC scores for the null model (assuming no bias//0, and for the final model of unisensory perception.
(XLSX)

S2 Table Unisensory model parameter estimates. Estimates of all unisensory model parameters *β*_0_, *β*_1*mT*_, *γ*_0*mT*_, and *γ*_1*mT*_, for modalities *m* visual and inertial, and *T* representing motion profile duration: 2, 4, or 6s.
(XLSX)

S3 Table Multisensory BIC scores. BIC scores for the MI, SS, and CI models, fitted either with invariant parameters for different motion profiles, or with different parameters for different conditions. The latter fits are marked with the subscript *T*.
(XLSX)

S4 Table Multisensory model parameters. Estimates of all multisensory model parameters.
(XLSX)

S1 Video Video of simulator motion. (AVI)

S1 Appendix Calculation of total variance. (PDF)

S1 Data Experimental data. (ZIP)
