# Supplementary material for: Causal Inference in Multisensory Heading Estimation
Source: PLoS One. 2017 Jan 6;12(1):e0169676. doi: 10.1371/journal.pone.0169676 (PMC5218471; doi:10.1371/journal.pone.0169676)
Supplement: S1 Fig — Plots of the bias in responses relative to visual heading angle (dots) as a function of discrepancy between visual and inertial heading angle. Dots with different colors represent data from different participants. The horizontal blue line indicates a perfect theoretical correspondence between responses and the visual heading angle; the diagonal orange line indicates a perfect theoretical correspondence between responses and the inertial heading angle. Discrepancy is defined as the angular difference between visual and inertial heading angle; bias is defined as the angular difference between the visual heading angle and the response. Respectively, the corresponding calculations are: Arg(eiθV/eiθI), and Arg(eiθV/eiθR). (PDF) [file pone.0169676.s001.pdf]

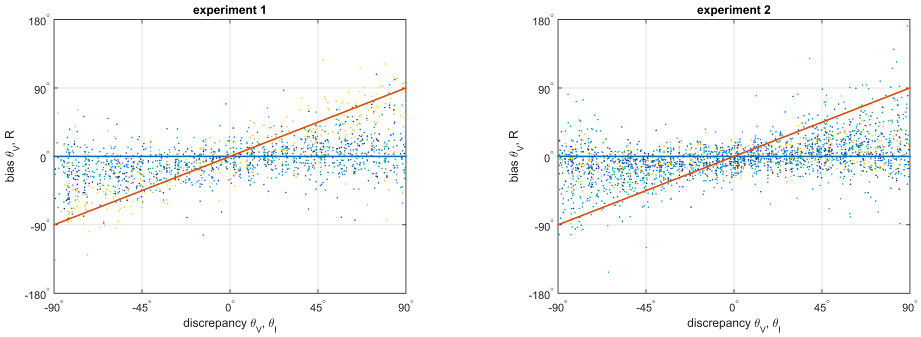

Fig. 1. Response bias as a function of discrepancy. Plots of the bias in responses relative to visual heading angle (dots) as a function of discrepancy between visual and inertial heading angle. Dots with different colors represent data from different participants. The horizontal blue line indicates a perfect theoretical correspondence between responses and the visual heading angle; the diagonal orange line indicates a perfect theoretical correspondence between responses and the inertial heading angle. Discrepancy is defined as the angular difference between visual and inertial heading angle; bias is defined as the angular difference between the visual heading angle and the response. Respectively, the corresponding calculations are: $\mathrm{Arg}(e^{i\theta_V}/e^{i\theta_I})$, and $\mathrm{Arg}(e^{i\theta_V}/e^{i\theta_R})$.

S1 Figure.
