# Supplementary material for: Causal Inference in Multisensory Heading Estimation
Source: PLoS One. 2017 Jan 6;12(1):e0169676. doi: 10.1371/journal.pone.0169676 (PMC5218471; doi:10.1371/journal.pone.0169676)
Supplement: S1 Appendix — (PDF) [file pone.0169676.s007.pdf]

S1 Appendix. We define a measure of total variance $\sigma^2_{tot.}$ as the integral of the circular variances σ^2_j of heading estimates over heading angles θ_j, ranging between $-\pi$ and π. The circular variance for a stimulus with heading angle θ_j is defined as

$$\sigma_j^2 = 1 - |\rho_j| \tag{1}$$

where ρ_j is the first trigonometric moment, $\rho_j = \mathrm{E}\left\{e^{i\theta_j}; \theta_j \sim f_{VM}(\mu, \kappa)\right\}$, which is calculated as

$$\rho_j = \int_{-\pi}^{\pi} e^{i\theta} f_{VM}(\theta; \mu, \kappa) d\theta \tag{1a}$$

where f_{VM} is the von Mises probability density function. This equation reduces to

$$\rho_j = \frac{e^{i\mu}\mathrm{I}_1(\kappa)}{\mathrm{I}_0(\kappa)}. \tag{1b}$$

I_n is the modified Bessel function of order n. This equation implies that

$$|\rho_j| = \frac{\mathrm{I}_1(\kappa)}{\mathrm{I}_0(\kappa)} \tag{1c}$$

We calculated the approximate integral of this measure over the range of j using the trapezoidal method 'trapz' in MATLAB (The MathWorks Inc., Natick, United States, version 2014b):

$$\sigma^2_{tot.} = \int_{-\pi}^{\pi} \left[1 - \frac{\mathrm{I}_1(\kappa(\theta_j, \gamma_0, \gamma_1))}{\mathrm{I}_0(\kappa(\theta_j, \gamma_0, \gamma_1))}\right] d\theta_j \tag{2}$$
